# Clinical and Virological Descriptive Study in the 2011 Outbreak of Dengue in the Amazonas, Brazil

**DOI:** 10.1371/journal.pone.0100535

**Published:** 2014-06-30

**Authors:** Valquiria do Carmo Alves Martins, Michele de Souza Bastos, Rajendranath Ramasawmy, Regina Pinto de Figueiredo, João Bosco Lima Gimaque, Wornei Silva Miranda Braga, Mauricio Lacerda Nogueira, Sergio Nozawa, Felipe Gomes Naveca, Luiz Tadeu Moraes Figueiredo, Maria Paula Gomes Mourão

**Affiliations:** 1 Fundação de Medicina Tropical Dr. Heitor Viera Dourado (FMT-HVD), Manaus, Amazonas, Brazil; 2 Universidade do Estado do Amazonas (UEA), Manaus, Amazonas, Brazil; 3 Universidade Nilton Lins, Manaus, Amazonas, Brazil; 4 Faculdade de Medicina de São Jose do Rio Preto (FAMERP), São Jose do Rio Preto, São Paulo, Brazil; 5 Instituto Leônidas & Maria Deane (ILMD), Fundação Oswaldo Cruz (FIOCRUZ), Manaus, Amazonas, Brazil; 6 Centro de Pesquisas em Virologia, Faculdade de Medicina de Ribeirão Preto (FMRP-USP), Ribeirão Preto, São Paulo, Brazil; University of Hong Kong, Hong Kong

## Abstract

**Background:**

Dengue is a vector-borne disease in the tropical and subtropical region of the world and is transmitted by the mosquito *Aedes aegypti*. In the state of Amazonas, Brazil during the 2011 outbreak of dengue all the four Dengue virus (DENV) serotypes circulating simultaneously were observed. The aim of the study was to describe the clinical epidemiology of dengue in Manaus, the capital city of the state of the Amazonas, where all the four DENV serotypes were co-circulating simultaneously.

**Methodology:**

Patients with acute febrile illness during the 2011 outbreak of dengue, enrolled at the Fundação de Medicina Tropical Dr. Heitor Viera Dourado (FMT-HVD), a referral centre for tropical and infectious diseases in Manaus, were invited to participate in a clinical and virological descriptive study. Sera from 677 patients were analyzed by RT-nested-PCRs for flaviviruses (DENV 1–4, Saint Louis encephalitis virus-SLEV, Bussuquara virus-BSQV and Ilheus virus-ILHV), alphavirus (Mayaro virus-MAYV) and orthobunyavirus (Oropouche virus-OROV).

**Principal Findings:**

Only dengue viruses were detected in 260 patients (38.4%). Thirteen patients were co-infected with more than one DENV serotype and six (46.1%) of them had a more severe clinical presentation of the disease. Nucleotide sequencing showed that DENV-1 belonged to genotype V, DENV-2 to the Asian/American genotype, DENV-3 to genotype III and DENV-4 to genotype II.

**Conclusions:**

Co-infection with more than one DENV serotype was observed. This finding should be warning signs to health authorities in situations of the large dispersal of serotypes that are occurring in the world.

## Introduction

Dengue virus (DENV) infection may display either benign acute febrile illness or severe disease such as dengue hemorrhagic fever/dengue shock syndrome (DHF/DSS) [1]. Over the last 31 years, Brazil has suffered successive outbreaks of dengue. The incidence of dengue continues to increase, and in the last decade 700,000 cases are reported per year. The mean age of severe dengue patients is decreasing. A high proportion of children are increasingly affected with severe disease [2]. In recent years, dengue outbreaks have displayed many atypical cases such as myocarditis, hepatitis, meningoencephalitis and acute kidney failure [3]. Fatality rates have also increased.

In Manaus, the capital city of the Amazonas state, circulation of the four DENV serotypes was observed during the 2011 outbreak and is the only city to date to report co-circulation of all four DENV serotypes simultaneously in Brazil, providing a clear evidence of dengue hyperendemicity [4]. The occurrence of severe forms of dengue is commonly related to secondary infections leading to antibody dependent enhancement (ADE) of DENV infection in macrophages [5]. Alternatively, severe forms of dengue could be related to virus mutants that emerge as part of a natural selection process [6]. Molecular epidemiology studies have shown that DENV genome suffers mutations and may lead each virus serotype to differentiate into multiple genotypes [7].

This study describes the clinical epidemiology of dengue in a city where all the four DENV serotypes are co-circulated simultaneously and also provides the opportunity to compare clinical severity to DENV serotypes. The results of the arboviruses surveillance performed by FMT-HVD during a dengue outbreak in 2011 are shown. Simultaneous circulations of the four DENV serotypes and different clades were observed. Dengue co-infection seems to lead to more severe manifestations.

## Materials and Methods

### Study design

This is a clinical and virological descriptive study to correlate clinical data with DENV serotypes during the 2011 outbreak of dengue in the Amazonas, Brazil. Patients with dengue were recruited from January 2011 through May 2011. A total of 677 patients from Manaus seeking medical assistance at the FMT-HVD agreed to participate in the study.

### Setting

All of the patients participating in this study are inhabitants of Manaus and were recruited at the FTM-HVD. The FMT-HVD, located in the Manaus city with almost a population of 2 million inhabitants, is the referral centre for acute febrile illness and tropical infectious diseases.

### Study sample

Patients with acute febrile illness but negative for malaria by thick blood smear test and presenting any two of the following symptoms in the first visit: headache, myalgia, arthralgia or malaise, were invited to participate in the present study. All of the patients participating in the study signed a written informed consent form. Parents or responsible party of children less than 18 years were required to sign a written inform consent form. This study was approved by the internal review board of the Ethics Committee of the FMT-HVD under the register number 2015.

### Inclusion criteria

Patients with acute febrile illness presenting fever less than 7 days were selected and classified according to the 2012 World Health Organization guidelines for dengue cases (http://www.who.int/denguecontrol/9789241504713/en/).

### Group I

#### Dengue without warning signs

Patients live in/travel to dengue endemic area, with fever and two of the following symptoms: nausea, vomiting, rash, aches and pains; tourniquet test positive; leukopenia.

### Group II

#### Dengue with warning signs

Patients with abdominal pain or tenderness; persistent vomiting; clinical fluid accumulation; mucosal bleed; lethargy, restlessness; liver enlargement >2 cm; increase in haematocrit concurrent with rapid decrease in platelet count.

### Group III

#### Severe dengue

Patients presenting severe plasma leakage leading to shock (DSS); fluid accumulation with respiratory distress; severe bleeding, and severe organ involvement including the liver (AST or ALT>1000 U/dL); the central nervous system (impaired consciousness); and the heart [8].

The clinical classification of the patients with dengue was performed by the clinicians according to the 2012 WHO guidelines for dengue on the day of enrollment. All of the severe dengue patients were hospitalized and followed-up until discharge. Furthermore, patients were seen in outpatient clinics 48 hours and 6–7 days after hospital discharge for hospitalized patients. Non-hospitalized patients were seen twice, between 48 hours and 6 days after first clinical assessment.

### Biochemical and hematology analysis

Blood sample was collected from all the patients on the day of presentation at the FMT-HVD. The biochemical tests were done with automatic analyzer CT 600i Wiener lab group apparatus while the hematological tests with the ABX pentra DX 120 HORIBA ABX Magnos bc.

### PCR screening

Five mL of blood was collected from all the participants on the day of presentation at the FMT-HVD. Serum was separated and RNA was purified from 200 µL using the QIAamp Viral RNA mini kit (Qiagen, Germany) according to the protocol of the kit. The extracted RNAs were stored at −70°C or immediately used for laboratory tests. cDNA was synthesized by reverse transcription (RT) with AccessQuick RT-PCR System kit (Promega, USA). The RT mixture consisted of 5 µl of RNA, 1 unit of the enzyme reverse transcriptase, 12.5 µl of AccessQuick Master Mix (2x), 1 µl of Random primer and 20 U of RNase inhibitor (RNaseOUT -Invitrogen, USA) in a final volume of 20 µl completed with RNase free water. The mixture was incubated at 72°C for 5 minutes (min) and at 45°C for 60 min and stored at −20°C until use.

For determining the DENV serotype, the cDNA were tested by a semi-nested multiplex reverse transcription-PCR as described elsewhere [9]. Briefly, each cDNA was subjected to polymerase chain reaction (PCR) amplification with D1 and D2 primers for 35 cycles: 1 min at 94°C, 1 min at 55°C, and 1 min at 72°C, with a final extension for 10 min at 72°C. A second round of amplification was conducted with 10 µl (diluted 1∶100) of the first reaction mixture, including DENV serotype-specific reverse primers (TS1–TS4), and the conserved forward primer D1. The same cycling parameters were used as in the first reaction.

All negative samples underwent another screening for DENV and other flaviviruses using the real time RT-PCR with Maxima SYBR GREEN/ROX reagents (Fermentas) [10]. Negative samples by real time RT-PCR were also tested by a conventional RT-PCR with *Flavivirus* genera specific primers followed by multiplex-nested-PCRs with species-specific primers [11]. After the first round of PCR with *Flavivirus* genera specific primers, nested-PCR primers specific for Dengue DENV 1–4, Saint Louis encephalitis virus-SLEV, Bussuquara virus-BSQV and Ilheus virus-ILHV, were used in the Multiplex-PCR. Precautions to avoid contamination were followed. Positive and negative controls were used in all reactions.

Samples previously negative to dengue were also tested for alphaviruses using genera-specific primers followed by a Multiplex-Nested-PCR with species-specific primers, including those for MAYV as previously described elsewhere [12]. For orthobunyaviruses, the samples were tested using genera-specific primers followed by a Nested-PCR with OROV specific primers [13].

All the samples that showed co-infections were reprocessed for RNA purifications from another aliquot of sera and undergone the RT-nested PCR for DENV to confirm co-infections and to avoid any doubt of contaminations.

### Nucleotide sequencing and phylogenetics

Nucleotide sequencing of amplicons was performed after treatment with Exonuclease I (20 U/µL) (BioLabs, New England) and Shrimp Alkaline Phosphatase-SAP (1 U/µL) (Fermentas). Briefly, the purification mixture included amplicons, 10 µL of Exo/SAP (0.025 µL SAP, 0.250 µL of Exonuclease I and 9.725 µL of Milli Q water). The mixture was incubated at 37°C for 30 min and 95°C for 5 min. Quantification of amplicons was performed in a NanoDrop 2000/2000c (Termo Scientific). Purified amplicons were directly sequenced using the Big dye Terminator Cycle Sequencing Kit (Applied Biosystems, EUA), following instructions of the manufacturer in the automatic sequencer ABI 3130L (Applied Biosystems, EUA).

Nucleotide sequences were analyzed by algorithms of the BioEdit Sequence Alignment software [14]. Nucleotide and putative amino acid sequences were compared to other sequences from GenBank for phylogenetic analysis by neighbor-joining method using the Mega 5.05 software with 1000 bootstrap replications [15].

### Gene Accession numbers

All the nucleotide sequences were submitted to GenBank and can be accessed with the following numbers: KF417476, KF417479, KF417480, KF417481, KF417478, KF417482, KF417477, KF417485, KF417484, KF417483, KF417488, KF417486, KF417489, KF417487, KF417490, KF417491, KF417496, KF417492, KF417493, KF417495, KF417494.

### Correlation of clinical data to DENV serotypes

Laboratory findings and DENV serotypes were crossed blindly to the clinical status of all of the patients.

### Statistical analysis

The Epi Info version 7.3.2 software was used for data handling. Descriptive analyses were performed to calculate means and standard deviation for continuous variable and relative frequency for categorical variable. Statistical analysis was performed using SPSS version 17.0. Chi-Square tests with Yates correction were performed for statistical comparison. Odds Ratio (OR) and 95% confidence interval (CI) were calculated by logistic regression to estimate the effect of co-infection or other clinical variables. Differences were considered significant at *p*≤0.05.

## Results

### General characteristics of the study population

A total of 677 patients with acute febrile illness and negative for malaria were included in the study. The mean age of the infected patients was 35.7±SD 15.7 (range 4 to 83 years) and 53.5% (139/260) were females.

### Molecular typing

Molecular typing for DENV showed positivity for 260 (38.4%) patients. The DENV serotypes were as follows: 46.2% (120/260) had DENV-2, 29.% (77/260) DENV-4, 10.0% (26/260) DENV-1 and 9.2% (24/260) DENV-3. Thirteen had co-infections: 2.3% (6/260) had DENV-2/4, 1.9% (5/260) DENV-1/4, 0.4% (1/260) DENV-1/2 and 0.4% DENV-3/4. All samples were negative to other flaviviruses, and also negative to MAYV and OROV. The patients were from different regions of the city and comparison among the regions to DENV serotypes did not show any difference. The co-infected patients were also from different regions.

Based on clinical presentation, dengue patients were classified as 192 dengue without warning signs cases, 45 severe dengue cases (DHF in 37 cases and 8 cases with neurological manifestations as somnolence, irritability and disorientation), and 23 dengue with warning signs cases. Clinical presentations of the dengue patients associated with DENV serotypes are shown in Table 1. Seven of the co-infected patients were classified as dengue with/without warning signs and the other six were hospitalized after initial evaluation. Co-infected patients with more than one DENV serotypes had more severe dengue (*p* = 0.004) compared to mono-infected patients. The serology and the viral load were not performed and it is possible that many of the co-infected patients may be of secondary infections and developed severe disease. Comparison of the different DENV serotypes with severity of the disease showed no statistical difference.

**Table 1:** Clinical presentation of dengue patients associated to infecting virus serotypes and co-infections.

Clinical presentation	DENV-1		DENV-2		DENV-3		DENV-4		Co-infections (DENV-1/2, DENV-1/4, DENV-2/4 and DENV-3/4)		Total
	N	%	N	%	N	%	N	%	N	%	
Dengue without warning signs	23	89	88	73	18	75	58	75	5	39	192
Dengue with warning signs	2	8	9	8	4	17	6	8	2	15	23
Severe dengue	1	4	23	19	2	8	13	17	6	46*	45
**Total**	**26**		**120**		**24**		**77**		**13**		**260**

*p = 0.004 Comparison of co-infected patients to mono-infected patients.

Clinical manifestations associated with dengue serotypes and co-infections are shown in Table 2. Cutaneous rash was more frequent (*p* = 0.003) among patients infected by DENV-1 or patients co-infected compared to patients infected with the other serotypes. Ocular pain (*p* = 0.03) and hemorrhagic phenomena (*p* = 0.01) were more frequent among patients co-infected with different serotype mixtures.

**Table 2:** Clinical aspects of dengue patients associated to the infecting virus serotype and co-infection.

Clinical presentation	DENV-1		DENV-2		DENV-3		DENV-4		Co-infection (DENV-1/2, DENV-1/4, DENV-2/4 and DENV-3/4)		*P* value
	N	%	N	%	N	%	N	%	N	%	
Headache	6	23	49	41	10	42	30	39	9	69	0.09
Myalgia	5	19	43	36	9	36	30	39	9	69	0.05
Arthralgia	2	8	28	23	5	21	18	23	7	54	0.03*
Rash	6	23	9	8	1	5	4	5	4	31	<0.01*
Ocular pain	4	15	28	23	9	38	15	20	7	54	0.03*
Alarm signs	3	12	32	27	7	29	19	25	8	62	0.02*
Hemorrhagic phenomena	1	4	21	18	5	21	10	13	8	62	<0.01*

*p<0.05 is considered statistically significant. Comparison of co-infected patients to mono-infected patients.

Laboratory findings associated to clinical severity of dengue are shown in Table 3. Levels of albumin in plasma (*p* = 0.03) and platelet counts <100.000/mm^3^ (*p*<0.01) were significantly related to severe dengue and dengue with warning signs respectively.

**Table 3:** Laboratory findings of dengue patients associated with classification of dengue severity.

Variable	Dengue without warning signs		Dengue with warning signs		Severe dengue		*P* Value
	N	%	N	%	N	%	
Hemoconcentration							
(45% for males, 42% for females)	46	36	7	35	23	51	0.74
Platelet <100×10^3^/µL	17	13	16	80	42	93	<0.01*
Leucocytes <3.0×10^3^/µL	36	29	2	29	2	17	0.66
Albumin <3.5 g/dL	4	4	1	5	6	14	0.03*

*p<0.05 is considered statistically significant. Comparisons between severe dengue patients to Dengue with/without warning signs patients.

Severe Dengue or minor hemorrhagic phenomena were prevalent among co-infected patients with an OR of 4.57 (*p* = 0.004) and 9.09 (*p*<0.001) respectively as shown in Table 4. Other variables such as arthralgia, rash, ocular pain, alarm signs, platelets counts and serum albumin level were also statistically significant compared to mono-infected patients. In table 5 is described the individual data for each patient co-infected with emphasis in their clinical and epidemiological findings.

**Table 4:** Dengue co-infection prevalence and associated variables.

Variable	N	N+ (%) (95%IC)	OR (95% CI)	*P* value
Total sample	260	13 (5.0) (2.4–7.6)	-	-
Severe dengue				
N	247	39 (15.8)	1	
Y	13	6 (46.20)	4.57 (1.43–14.33)	0.004
Arthralgia				
N	247	53 (21.5)	1	
Y	13	7 (53.8)	4.27 (1.38–13.25)	0.013
Rash				
N	247	20 (8.1)	1	
Y	13	4 (30.8)	5.04 (1.43–17.84)	0.023
Ocular pain				
N	247	56 (27.7)	1	
Y	13	7 (53.8)	3.98 (1.28–12.32)	0.018
Hemorrhagic phenomena				
N	247	37 (15.0)	1	
Y	13	8 (61.5)	9.08 (2.82–29.28)	<0.001
Alarm signs				
N	247	61 (24.7)	1	
Y	13	8 (61.5)	4.88 (1.54–15.47)	0.007
Platelet <100×10^3^/µL				
N	182	66 (36.3)	1	
Y	11	9(81.1)	7.91 (1.66–37.71)	0.004
Albumin <3.5 g/dL				
N	144	8 (5.6)	1	
Y	11	3 (27.3)	6.37 (1.41–28.75)	0.032

Odds Ratio (OR) were calculated by Chi-squared test.

**Table 5:** Clinical presentation of dengue in co-infected patients.

Patient	Age/Gender	Days of fever	Clinical diagnosis	Hospitalization	Dengue serotype (PCR)	Hemorrhagic events	Cavitary effusion	Abdominal pain	Gallbladder wall thickening
1	25/M	3	I	No	**DENV-1/DENV-4**	N	N	ND	ND
2	31/M	ND	I	No	**DENV-1/DENV-2**	N	N	ND	ND
3	17/F	5	II	Yes	**DENV-2/DENV-4**	Y	N	N	Y
4	64/F	7	III	Yes	**DENV-3/DENV-4**	Y	N	Y	N
5	38/M	3	I	Yes	**DENV-1/DENV-4**	Y	N	N	N
6	27/M	4	III	Yes	**DENV-2/DENV-4**	Y	Y	Y	Y
7	18/F	6	III	Yes	**DENV-1/DENV-4**	Y	N	Y	N
8	61/F	4	III	Yes	**DENV-2/DENV-4**	N	N	N	N
9	45/F	ND	I	No	**DENV-1/DENV-4**	N	N	ND	ND
10	36/F	ND	I	No	**DENV-2/DENV-4**	N	N	ND	ND
11	28/F	7	III	Yes	**DENV-1/DENV-4**	Y	N	N	Y
12	17/F	4	II	Yes	**DENV-2/DENV-4**	Y	N	N	N
13	40/M	6	III	Yes	**DENV-2/DENV-4**	Y	Y	Y	N

I: dengue without warning signs; II: dengue with warning signs; III: severe dengue; M: male; F: female; Y: yes; N: No; ND: no data.

### Phylogenetics analysis

**Figure 1:**
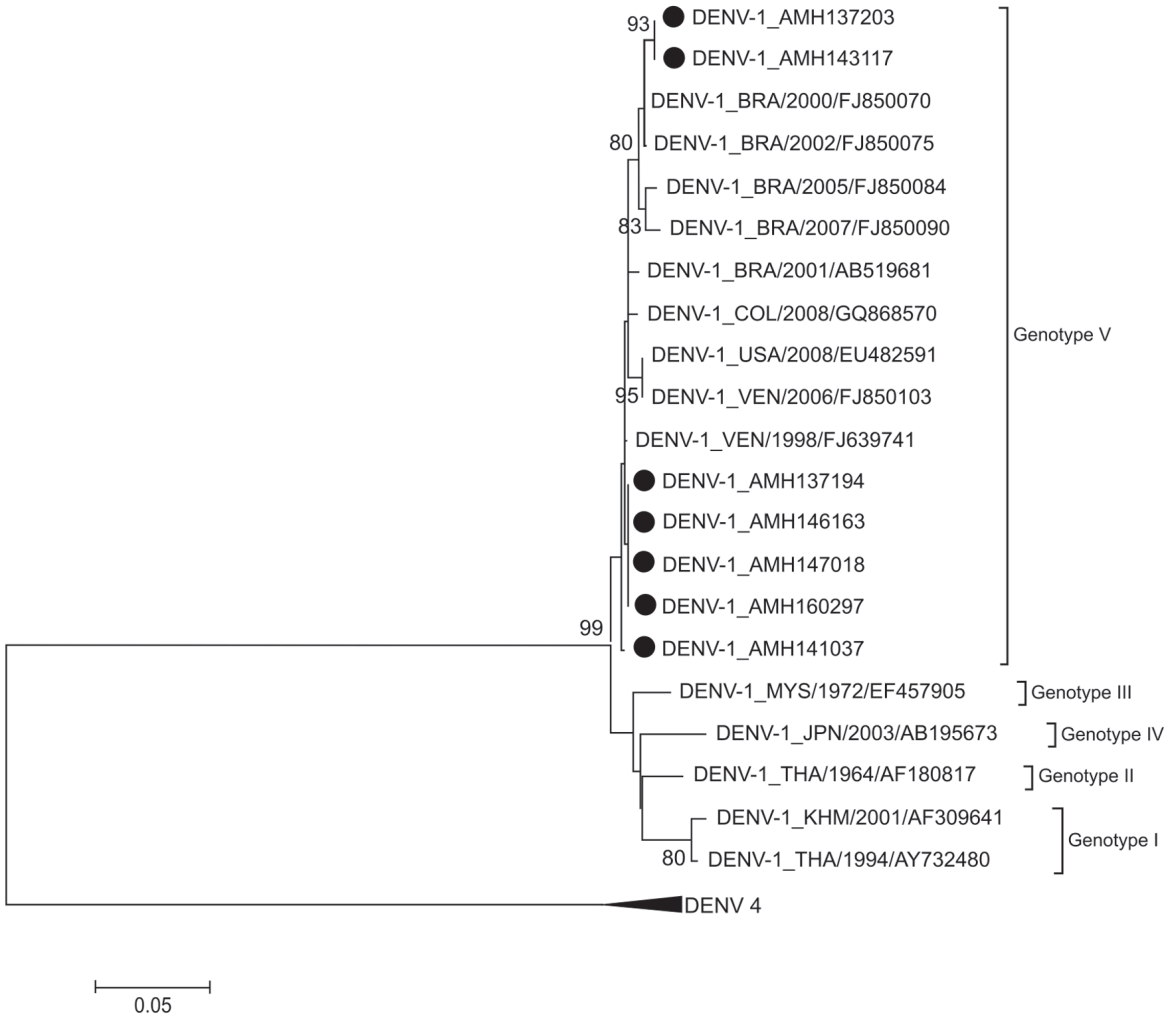
Phylogenetic tree of DENV-1. The neighbor-joining tree was constructed based on the prM-C genome region and sequences were compared to sequences retrieved from Gene-Bank (NCBI, USA). Sequences from this study are marked with dots. Only boot-strap values above 75% are shown in the figure.

**Figure 2:**
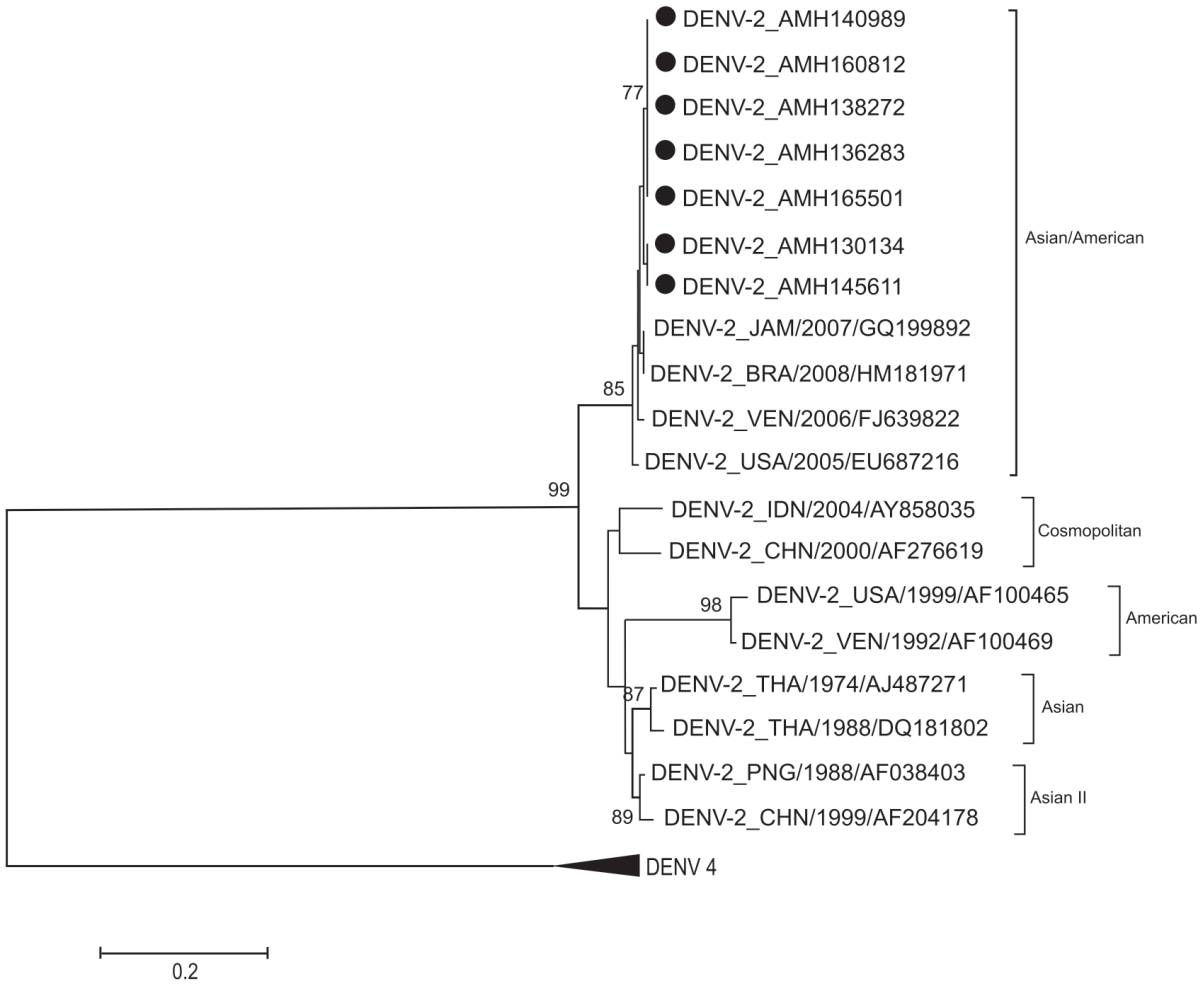
Phylogenetic tree of DENV-2. The neighbor-joining tree was constructed based on the prM-C genome region and sequences were compared to sequences retrieved from Gene-Bank (NCBI, USA). Sequences from this study are marked with dots. Only boot-strap values above 75% are shown in the figure.

**Figure 3:**
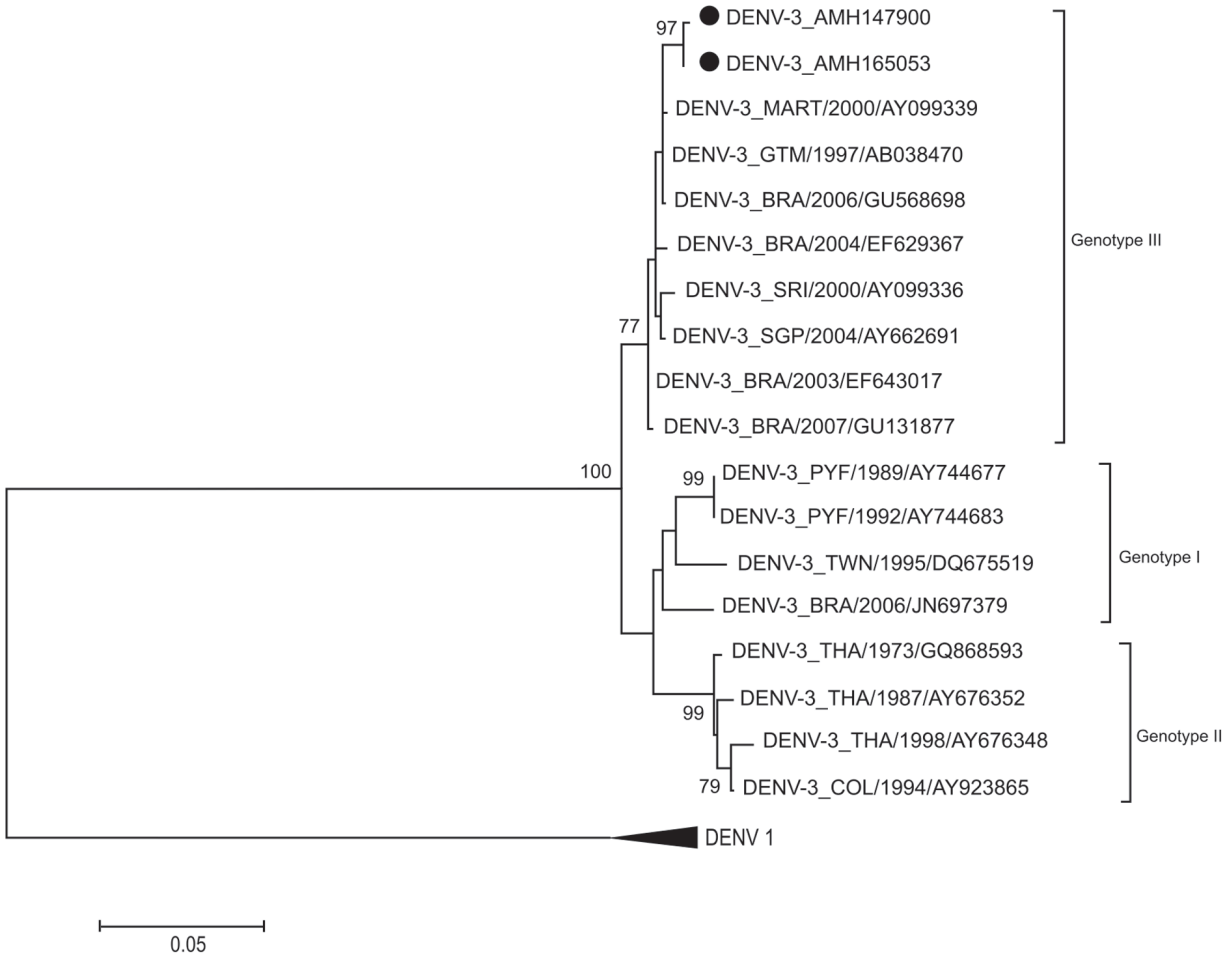
Phylogenetic tree of DENV-3. The neighbor-joining tree was constructed based on the prM-C genome region and sequences were compared to sequences retrieved from Gene-Bank (NCBI, USA). Sequences from this study are marked with dots. Only boot-strap values above 75% are shown in the figure.

**Figure 4:**
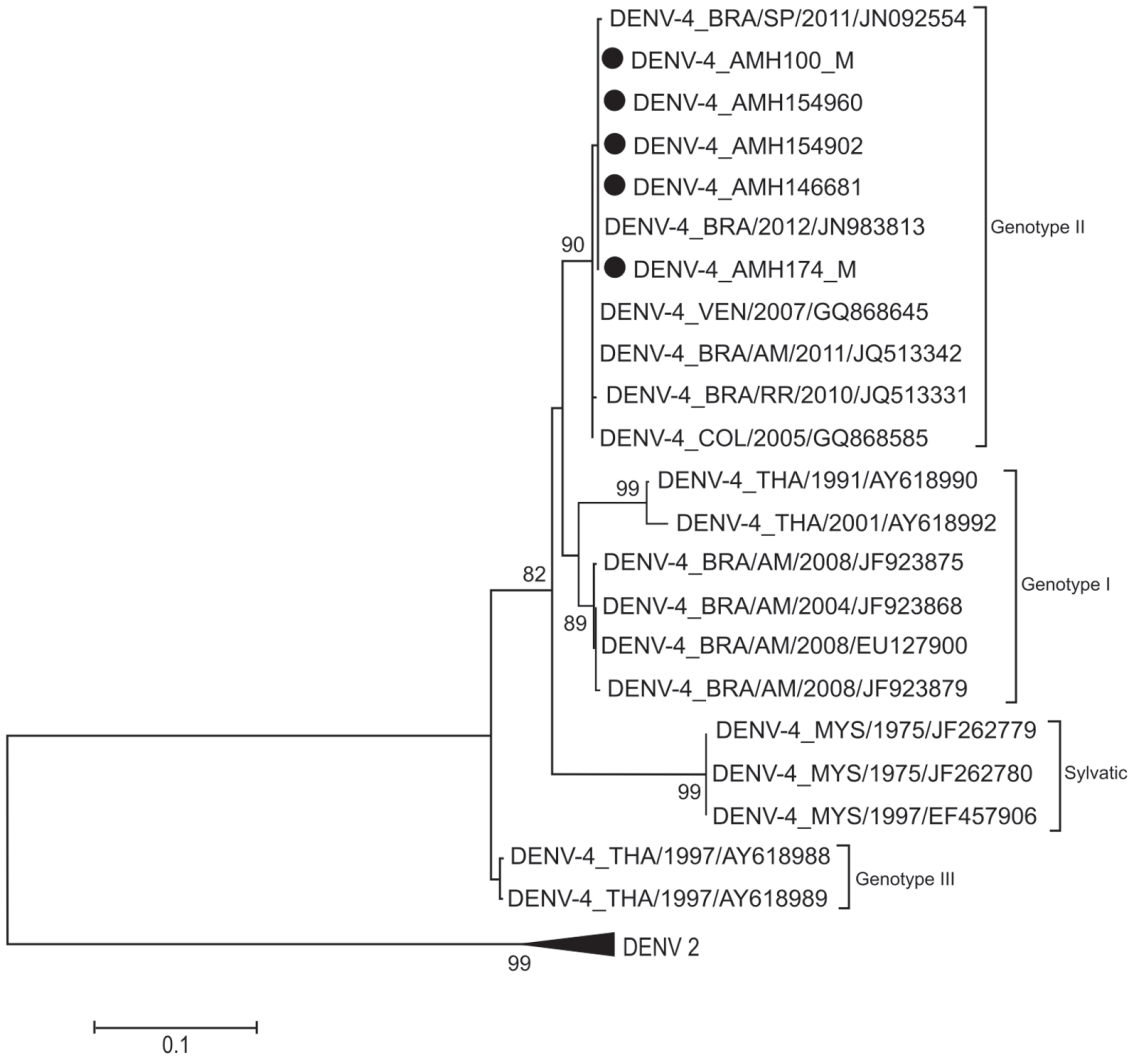
Phylogenetic tree of DENV-4. The neighbor-joining tree was constructed based on the prM-C genome region and sequences were compared to sequences retrieved from Gene-Bank (NCBI, USA). Sequences from this study are marked with dots. Only boot-strap values above 75% are shown in the figure.

Nucleotides sequencing of a fragment of 453 base pairs for the region of prM-C of 21 samples (16 cases with dengue without warning signs, 3 with severe dengue and 2 with dengue with warning signs) were performed. Of the 21 samples, 7 were of DENV-1, 7 of DENV-2, 2 of DENV-3 and 5 of DENV-4. Nucleotide sequences were aligned and neighbor joining phylogenetic trees were generated for each DENV serotype, as shown at Figures 1, 2, 3 and 4. DENV-1 (Fig.1), DENV-2 (Fig.2), DENV-3 (Fig.3) and DENV-4 (Fig. 4) belong to the genotype V, Asian/American genotype, genotype III and genotype II respectively. All the nucleotide sequences were submitted to NCBI and the corresponding GenBank accession numbers were generated (Table S1, ALVES VCR 301013 STROBE).

## Discussion

In the present study, 677 patients with suspected dengue fever were evaluated and through three different molecular techniques only DENV were detected in 260 patients. All the four DENV serotypes were detected with the predominance of DENV-2 and DENV-4. Thirteen patients were co-infected with more than one DENV serotypes. The co-infected patients manifested severe dengue and dengue with warning signs compare to mono-infected patients. The clinical presentation of dengue without warning signs among co-infected patients was exuberant with cutaneous rash, arthralgia and ocular pain.

No difference was observed between the DENV serotypes and severity of the disease. This is an important finding as DENV-2 in Brazil is related to severe disease [16], [17]. Co-infection with more than one DENV serotypes has been gradually reported in the last decade. Rocco and colleagues (1998) were the first to report a case of co-infection by serotypes of DENV-1 and 2 in a 30-year-old resident of Miranda, MS, Brazil in 1996 and Lorono-Pino et al (1999) suggested that infections with more than one serotype should be excepted in hyperendemic areas [18], [19]. Since then several other authors have been reporting DENV co-infections [20]–[22].

In this study, co-infections were observed in 13 patients. Different from three previous studies in Brazil where all the six co-infected reported patients were of dengue without warning signs [18], [23], [24], 61% (8/13) of the co-infected patients in this study had either severe dengue or dengue with warning signs. Some studies have shown that co-infection can cause severe disease. In a study conducted in India, six out of nine co-infected patients had DHF [25].

Altogether it can be suggested that concurrent infections by more than one serotype could influence the clinical expression of dengue. The presence of two DENV serotypes in an individual may possibly increase the DENV viremia levels compare to a mono-infected patient. The number of infected leucocytes in co-infected patients may be higher and can induce a large cytotoxicity phenomena leading to a cytokine storm and progress to severe disease. High viral load and secondary infections may also trigger the development of severe disease. The drawback in our study is that the serology and the viral load were not performed. It cannot be rule out that many of the patients were of secondary infections and developed severe disease.

DENV can cause manifestations of central nervous system and an increasing number of cases have been reported in the last few years [26]–[29]. In the present study, among 45 patients with severe dengue, eight had neurologic alteration (alteration in consciousness) and were not associated to any DENV serotype or co-infections. All of the patients with neurologic alteration recovered from the disease. These findings underscore the need to investigate dengue in patients with viral meningoencephalitis in Manaus and other endemic areas.

Haematocrit, a parameter associated with capillary leak syndrome, was not significantly increased among patients with severe dengue and dengue with warning signs. It is possible that the oral rehydration therapy offered to all these patients has been started before installation of a marked capillary leak. This intervention certainly has reduced severe cases (only 1% among our patients) and fatalities. However, platelet counts under 100.000/mm^3^ were related to severe dengue patients and to dengue with warning signs.

Manaus is relatively isolated from the rest of the country. To identify whether the DENV genotypes that are circulating in the country are similar in Manaus, nucleotide sequencing of the selected samples were performed. All the DENV-1 serotypes were of genotype V, the only genotype detected so far in Brazil [30]. The phylogenetic tree of DENV-1 (Fig. 1) displayed two distinct clades of genotype V from patients in this study, which is supported by a high bootstrap value. In our samples DENV genotypes and clades were not associated with severity of the disease and neither to any region of the city. However, the dataset is small and need to be further characterized to confirm this observation. In fact the co-circulation of different clades and/or clade replacement is related to high genetic diversity of that isolates and can lead to new distinct biological proprieties that may induce more severe disease. Recently, two complete distinct strains (both belonging to genotype V) of DENV-1 were observed in Brazil [30].

DENV-2 serotypes from Manaus were grouped with the Asian/American genotype that is more virulent than the indigenous American genotype and is also the predominant genotype in Brazil [16], [31]. DENV-3 belonged to genotype III introduced from the Caribbean Islands and present in Brazil since the last 15 years [32], [33]. All DENV-4 analyzed in this study belonged to genotype II. DENV-4 was first described in Manaus in 2008 [34]. It was found that this DENV-4 belonged to the genotype I, which is of Asian origin and was never described in the American Continent [34], [35]. Presently, probably, 2 genotypes (I and II) of DENV-4 are circulating in Manaus [36], [37].

## Conclusions

The co-circulation of multiple serotypes or strains of dengue genotypes are becoming common in endemic region and may increase the risk of major epidemics as observed in Manaus during the 2011 outbreak where more than 50,000 cases of dengue virus infections were reported. Future studies with quantification of viruses and serology test will be needed to understand the mechanism of how co-infection with more than one DENV serotypes may lead to severe dengue disease in the city of Manaus.

## Supporting Information

Table S1 **Nucleotide sequences used for generating phylogenetic trees are registered in GenBank (NCBI, USA).** ALVES VCR 301013 STROBE.
(DOC)

## References

[1] Gubler DJ (2002) The global emergence/resurgence of arboviral diseases as public health problems. Archives of medical research 33: 330–342. 12234522 10.1016/s0188-4409(02)00378-8

[2] Rocha LA, Tauil PL (2009) [Dengue in children: clinical and epidemiological characteristics, Manaus, State of Amazonas, 2006 and 2007]. Rev Soc Bras Med Trop 42: 18–22. 19287930 10.1590/s0037-86822009000100005

[3] Figueiredo LTM (2012) Dengue in Brazil. Revista da Sociedade Brasileira de Medicina Tropical 45: 285–285. 22760122 10.1590/s0037-86822012000300001

[4] Bastos MS, Figueiredo RM, Ramasawmy R, Itapirema E, Gimaque JB, et al (2012) Simultaneous circulation of all four dengue serotypes in Manaus, State of Amazonas, Brazil in 2011. Rev Soc Bras Med Trop 45: 393–394. 22760143 10.1590/s0037-86822012000300022

[5] Halstead SB (1988) Pathogenesis of dengue: challenges to molecular biology. Science 239: 476–481. 3277268 10.1126/science.3277268

[6] Rico-Hesse R, Harrison LM, Salas RA, Tovar D, Nisalak A, et al (1997) Origins of dengue type 2 viruses associated with increased pathogenicity in the Americas. Virology 230: 244–251. 9143280 10.1006/viro.1997.8504

[7] Wu W, Bai Z, Zhou H, Tu Z, Fang M, et al (2011) Molecular epidemiology of dengue viruses in southern China from 1978 to 2006. Virol J 8: 322. 21703015 10.1186/1743-422X-8-322 PMC3138434

[8] WHO/TDR (2012) Handbook for clinical management of dengue. Geneva, Switzerland World Health Organization. pp. 124.

[9] Lanciotti RS, Calisher CH, Gubler DJ, Chang GJ, Vorndam AV (1992) Rapid detection and typing of dengue viruses from clinical samples by using reverse transcriptase-polymerase chain reaction. Journal of clinical microbiology 30: 545–551. 1372617 10.1128/jcm.30.3.545-551.1992 PMC265106

[10] Chao DY, Davis BS, Chang GJ (2007) Development of multiplex real-time reverse transcriptase PCR assays for detecting eight medically important flaviviruses in mosquitoes. Journal of clinical microbiology 45: 584–589. 17108075 10.1128/JCM.00842-06 PMC1829073

[11] de Morais Bronzoni RV, Baleotti FG, Ribeiro Nogueira RM, Nunes M, Moraes Figueiredo LT (2005) Duplex reverse transcription-PCR followed by nested PCR assays for detection and identification of Brazilian alphaviruses and flaviviruses. Journal of clinical microbiology 43: 696–702. 15695666 10.1128/JCM.43.2.696-702.2005 PMC548032

[12] Bronzoni RV, Moreli ML, Cruz AC, Figueiredo LT (2004) Multiplex nested PCR for Brazilian Alphavirus diagnosis. Transactions of the Royal Society of Tropical Medicine and Hygiene 98: 456–461. 15186933 10.1016/j.trstmh.2003.09.002

[13] Moreli ML, Aquino VH, Cruz AC, Figueiredo LT (2002) Diagnosis of Oropouche virus infection by RT-nested-PCR. Journal of medical virology 66: 139–142. 11748670 10.1002/jmv.2122

[14] Hall TA (1999) BioEdit: a user-friendly biological sequence alignment editor and analysis program for Windows 95/98/NT. Nucl Acids Symp Ser 41: 95–98.

[15] Tamura K, Peterson D, Peterson N, Stecher G, Nei M, et al (2011) MEGA5: molecular evolutionary genetics analysis using maximum likelihood, evolutionary distance, and maximum parsimony methods. Mol Biol Evol 28: 2731–2739. 21546353 10.1093/molbev/msr121 PMC3203626

[16] Drumond BP, Mondini A, Schmidt DJ, Bronzoni RV, Bosch I, et al (2013) Circulation of different lineages of dengue virus 2, genotype american/asian in Brazil: dynamics and molecular and phylogenetic characterization. PLoS One 8: e59422. 23533624 10.1371/journal.pone.0059422 PMC3606110

[17] Oliveira MF, Galvao Araujo JM, Ferreira OC Jr, Ferreira DF, Lima DB, et al (2010) Two lineages of dengue virus type 2, Brazil: Emerg Infect Dis. 2010 Mar 16(3): 576–8 doi: 10.3201/eid1603.090996 10.3201/eid1603.090996 PMC3322019 20202456

[18] Rocco IM, Barbosa ML, Kanomata EH (1998) Simultaneous infection with dengue 1 and 2 in a Brazilian patient. Rev Inst Med Trop Sao Paulo 40: 151–154. 9830728 10.1590/s0036-46651998000300004

[19] Lorono-Pino MA, Cropp CB, Farfan JA, Vorndam AV, Rodriguez-Angulo EM, et al (1999) Common occurrence of concurrent infections by multiple dengue virus serotypes. Am J Trop Med Hyg 61: 725–730. 10586902 10.4269/ajtmh.1999.61.725

[20] Araújo FMdC, Nogueira RMR, Araújo JMGd, Ramalho ILC, Roriz MLFdS, et al (2006) Concurrent infection with dengue virus type-2 and DENV-3 in a patient from Ceará, Brazil. Memórias do Instituto Oswaldo Cruz 101: 925–928. 17293990 10.1590/s0074-02762006000800017

[21] Wenming P, Man Y, Baochang F, Yongqiang D, Tao J, et al (2005) Simultaneous infection with dengue 2 and 3 viruses in a Chinese patient return from Sri Lanka. J Clin Virol 32: 194–198. 15722024 10.1016/j.jcv.2004.04.010

[22] Yu M, Peng WM, Fan BC, Deng YQ, Qin ED (2004) [Demonstration of simultaneous infection with dengue type 2 and 3 in a Chinese patient]. Wei Sheng Wu Xue Bao 44: 717–719. 16110945

[23] Figueiredo RM, Naveca FG, Oliveira CM, Bastos Mde S, Mourao MP, et al (2011) Co-infection of Dengue virus by serotypes 3 and 4 in patients from Amazonas, Brazil. Rev Inst Med Trop Sao Paulo 53: 321–323. 22183455 10.1590/s0036-46652011000600004

[24] dos Santos CL, Bastos MA, Sallum MA, Rocco IM (2003) Molecular characterization of dengue viruses type 1 and 2 isolated from a concurrent human infection. Rev Inst Med Trop Sao Paulo 45: 11–16. 12751316 10.1590/s0036-46652003000100003

[25] Bharaj P, Chahar HS, Pandey A, Diddi K, Dar L, et al (2008) Concurrent infections by all four dengue virus serotypes during an outbreak of dengue in 2006 in Delhi, India. Virol J 5: 5–1. 18182120 10.1186/1743-422X-5-1 PMC2253528

[26] Soares CN, Cabral-Castro MJ, Peralta JM, Freitas MR, Puccioni-Sohler M (2010) Meningitis determined by oligosymptomatic dengue virus type 3 infection: report of a case. Int J Infect Dis 14: e150–152. 19501535 10.1016/j.ijid.2009.03.016

[27] Soares CN, Faria LC, Peralta JM, de Freitas MR, Puccioni-Sohler M (2006) Dengue infection: neurological manifestations and cerebrospinal fluid (CSF) analysis. J Neurol Sci 249: 19–24. 16870213 10.1016/j.jns.2006.05.068

[28] Domingues RB, Kuster GW, Onuki-Castro FL, Souza VA, Levi JE, et al (2008) Involvement of the central nervous system in patients with dengue virus infection. J Neurol Sci 267: 36–40. 17959198 10.1016/j.jns.2007.09.040

[29] Misra UK, Kalita J, Syam UK, Dhole TN (2006) Neurological manifestations of dengue virus infection. J Neurol Sci 244: 117–122. 16524594 10.1016/j.jns.2006.01.011

[30] Drumond BP, Mondini A, Schmidt DJ, Bosch I, Nogueira ML (2012) Population dynamics of DENV-1 genotype V in Brazil is characterized by co-circulation and strain/lineage replacement. Arch Virol 157: 2061–2073. 22777179 10.1007/s00705-012-1393-9

[31] Bona AC, Twerdochlib AL, Navarro-Silva MA (2012) Genetic diversity of dengue virus serotypes 1 and 2 in the State of Parana, Brazil, based on a fragment of the capsid/premembrane junction region. Rev Soc Bras Med Trop 45: 297–300. 22760124 10.1590/s0037-86822012000300003

[32] Alfonso HL, Amarilla AA, Goncalves PF, Barros MT, Almeida FT, et al (2012) Pylogenetic relationship of dengue virus type 3 isolated in Brazil and Paraguay and global evolutionary divergence dynamics. Virol J 9: 124. 22716071 10.1186/1743-422X-9-124 PMC3494512

[33] Aquino VH, Anatriello E, Goncalves PF, Da Silva EV, Vasconcelos PF, et al (2006) Molecular epidemiology of dengue type 3 virus in Brazil and Paraguay, 2002–2004. Am J Trop Med Hyg 75: 710–715. 17038699

[34] Figueiredo RM, Naveca FG, Bastos MS, Melo MN, Viana SS, et al (2008) Dengue virus type 4, Manaus, Brazil. Emerging infectious diseases 14: 667–669. 18394292 10.3201/eid1404.071185 PMC2570911

[35] de Melo FL, Romano CM, de Andrade Zanotto PM (2009) Introduction of dengue virus 4 (DENV-4) genotype I into Brazil from Asia? PLoS neglected tropical diseases 3: 28. 10.1371/journal.pntd.0000390 PMC2669502 19399169

[36] de Figueiredo ML, Alfonso HL, Amarilla AA, Figueiredo LT, Aquino VH, et al (2013) Detection of DENV-4 genotype I from mosquitoes collected in the city of Manaus, Brazil. Virol J 10: 60. 23421733 10.1186/1743-422X-10-60 PMC3599326

[37] Naveca FG, Souza VC, Silva GA, Maito RM, Granja F, et al (2012) Complete genome sequence of a Dengue virus serotype 4 strain isolated in Roraima, Brazil. J Virol 86: 1897–1898. 22247521 10.1128/JVI.06731-11 PMC3264359

