# Supplementary material for: Clinical and Virological Descriptive Study in the 2011 Outbreak of Dengue in the Amazonas, Brazil
Source: PLoS One. 2014 Jun 30;9(6):e100535. doi: 10.1371/journal.pone.0100535 (PMC4076277; doi:10.1371/journal.pone.0100535)
Supplement: Table S1 — Nucleotide sequences used for generating phylogenetic trees are registered in GenBank (NCBI, USA). ALVES VCR 301013 STROBE. (DOC) [file pone.0100535.s001.doc]

Supplementary Table S1. Nucleotide sequences are registered in GenBank (NCBI, USA).

| **GenBank** | **Description of the sample** | **Genotype** | **Clinical presentation** | **% REF** | **gap** |
| --- | --- | --- | --- | --- | --- |
| KF417476 | DENV-1_AMH137194 | 1-V | I | 98 | 0 |
| KF417479 | DENV-1_AMH137203 | 1-V | I | 97.5 | 0 |
| KF417480 | DENV-1_AMH141037 | 1-V | I | 100 | 0 |
| KF417481 | DENV-1_AMH143117 | 1-V | I | 100 | 0 |
| KF417478 | DENV-1_AMH146163 | 1-V | I | 98.7 | 0 |
| KF417482 | DENV-1_AMH147018 | 1-V | II | 100 | 0 |
| KF417477 | DENV-1_AMH160297 | 1-V | III | 100 | 0 |
| KF417485 | DENV-2_AMH136283 | 2-AsianAmerican | I | 99.3 | 0 |
| KF417484 | DENV-2_AMH138272 | 2-AsianAmerican | I | 98.7 | 0 |
| KF417483 | DENV-2_AMH130134 | 2-AsianAmerican | I | 98.7 | 0 |
| KF417488 | DENV-2_AMH140989 | 2-AsianAmerican | I | 98.6 | 0 |
| KF417486 | DENV-2_AMH145611 | 2-AsianAmerican | I | 98.2 | 0 |
| KF417489 | DENV-2_AMH160812 | 2-AsianAmerican | III | 99.8 | 0 |
| KF417487 | DENV-2_AMH165582 | 2-AsianAmerican | I | 100 | 0 |
| KF417490 | DENV-3_AMH147900 | 3-III | I | 97.1 | 0 |
| KF417491 | DENV-3_AMH165053 | 3-III | I | 99.3 | 0 |
| KF417496 | DENV-4_AMH146681 | 4-II | I | 89.6 | 0 |
| KF417492 | DENV-4_AMH154902 | 4-II | I | 100 | 0 |
| KF417493 | DENV-4_AMH154960 | 4-II | I | 99.6 | 0 |
| KF417495 | DENV-4_AMH100_M | 4-II | III | 97.7 | 0 |
| KF417494 | DENV-4_AMH174_M | 4-II | II | 99.6 | 0 |

I: dengue without warning signs; II: dengue with warning signs; III: severe dengue;

% REF: Percentage of nucleotide sequence similar to reference sequences; gap means if sequences were contiguous or not;
